# Prophylactic and Remedy in Preliminary Diarrhœas and Vomitings Present in Choleraic Disease

**Published:** 1873-09-01

**Authors:** Thos. Bevan

**Affiliations:** 11 Harmon Court


Editors Medical Examiner :

*Gentlemen :* — On the basis of the German theory, or zymotic origin of choleraic disease, I would suggest the following formulæ, for administration as a prophylactic and remedy in the preliminary diarrhœas and vomitings present in attacks of that malady :

℞.—Sulphurous acid, (50 vols. gas—U. S. P.) ℥ ii.

Sulphite magnesia, ʒ ii.

Tinct. capsicum, ʒ iv.

Aquæ destill, ℥ ii.—Mix.

A teaspoonful, in three or four ounces of cold water, two or three times a day, as *preventive.*

℞.—Sulphurous acid, (50 vols. gas—U. S. P.) ℥ ii.

Sulphite magnesia, ʒ ii.

Sulph. morph. gr. ii.

Tinct. capsicum, ʒ iv.

Aquæ destill, ℥ ii.—Mix.

A teaspoonfull every half hour or hour, in the preliminary diarrhœa also diluted with a little cold water, as *remedy.*

The principle on which this prophylactic and treatment is based, is that of the extension to the human organism itself of the influence of agents that are well known to act as tonics to the lower forms of organic life —spores, fungi, vibriones, lacteria, etc., etc. In the "remedy" is found simply the addition of the sixteenth of a grain of morphia, which, in this dose, acts only as a *vaso-motor* stimulant, never as narcotic sedative. If you get somewhat lessened peristaltic movement, at the same time that the germs are destroyed in the digestive passages by the antzymotics, there can be no objection to the re-absorption of the effused serum; in fact, with the greatly diminished vascular tension, and diminished fluidity of the blood, *en masse*, it is a positive help to your patient. The magnesia may be counted on as a decidedly favoring elimination, in addition to its antizymotic effects. The tinct. capsicum will favor absorption by its stimulant action on the alimentary mucous membranes, while the sulphurous acid, in addition to its antizymotic effects, is a reliable styptic astringent. This mixture is well borne by the stomach; and it is believed by the writer that, in the presence of the choleraic epidemic constitution, an anti-germinative saturation may be maintained for long periods of time, without detriment to the healthful functionment of the economy. A fair test of its merits is invited by all interested in sanitary science.

I am, etc., yours truly,
THOS. BEVAN.
11 Harmon Court, Aug. 20, '73.

