# Correction

**Published:** 1873-09-15

**Authors:** 


CORRECTION.—In the letter of Prof. Thos. Bevan, published in the last number of THE EXAMINER, there occurred several errors, overlooked in the proof-reading. In the first line, *Germ*, not *German theory*, was intended. At the bottom of the page, *tonics* should read *toxic*, and *lacteria*, *bacteria*.

